# Supplementary material for: Externally induced frontoparietal synchronization modulates network dynamics and enhances working memory performance
Source: eLife. 2017 Mar 14;6:e22001. doi: 10.7554/eLife.22001 (PMC5349849; doi:10.7554/eLife.22001)
Supplement: Supplementary file 1. — For each cluster, the peak and five local maxima within the cluster are listed along with x-y-z locations in MNI space. R = right hemisphere; L = left hemisphere. DOI: http://dx.doi.org/10.7554/eLife.22001.018 [file elife-22001-supp1.docx]

**Supplementary File 1**

**Table supplement 1 (related to Figure 4A).** Results from the whole-brain analysis for the contrast (tACS 0° > tACS OFF) for the 2-back task (Cluster corrected Z = 2.3, p < 0.05, n=21).

|  | | **Region** | **Cluster size** | **Z Peak** | **MNI (peak)** | | |
| --- | --- | --- | --- | --- | --- | --- | --- |
|  |  |  |  |  | **X** | **Y** | **Z** |
| **tACS 0° > tACS OFF** | | | | | | | |
|  | R | Parietal Lobe; Supramarginal gyrus | 5891 | 4.09 | 66 | -40 | 24 |
|  | L | Lateral occipital cortex (superior division) |  | 4.00 | -34 | -68 | 16 |
|  | L | Occipital pole |  | 3.96 | -26 | -96 | -16 |
|  | L | Occipital pole |  | 3.92 | -24 | -92 | -12 |
|  | L | Lateral occipital cortex (inferior division) |  | 3.83 | -52 | -78 | -6 |
|  | R | Temporal occipital fusiform cortex |  | 3.76 | 42 | -58 | -10 |
|  | R | Thalamus | 3082 | 3.86 | 16 | -4 | 12 |
|  | R | Caudate |  | 3.78 | 20 | 0 | 22 |
|  | R | Caudate |  | 3.74 | 20 | 14 | 12 |
|  | R | Caudate |  | 3.71 | 16 | -2 | 24 |
|  | R | Precentral gyrus |  | 3.69 | 62 | 10 | 30 |
|  | R | Thalamus | 1068 | 3.66 | 4 | -12 | 14 |
|  | L | Supramarginal gyrus |  | 3.49 | -54 | -48 | 42 |
|  | L | Supramarginal gyrus; superior parietal lobule |  | 3.47 | -40 | -46 | 34 |
|  | L | Supramarginal gyrus |  | 3.40 | -50 | -50 | 42 |
|  | L | Supramarginal gyrus |  | 3.26 | -58 | -46 | 38 |
|  | L | Supramarginal gyrus |  | 3.22 | -64 | -44 | 34 |
|  | L | Angular gyrus |  | 3.20 | -40 | -54 | 44 |
|  | R | Precentral gyrus; Middle frontal gyrus | 597 | 3.32 | 38 | -6 | 58 |
|  | R | Precentral gyrus |  | 3.12 | 44 | -2 | 62 |
|  | R | Middle frontal gyrus |  | 2.82 | 52 | 10 | 54 |
|  | R | Middle frontal gyrus |  | 2.81 | 54 | 8 | 50 |
|  | R | Precentral gyrus; Middle frontal gyrus |  | 2.81 | 42 | 0 | 48 |
|  | R | Precentral gyrus; Postcentral gyrus |  | 2.77 | 58 | -6 | 42 |

For each cluster, the peak and 5 local maxima within the cluster are listed along with x-y-z locations in MNI space.

No clusters were observed for the contrast (tACS OFF > tACS 0°).

R = right hemisphere; L = left hemisphere

**Table supplement 2 (related to Figure 4A).** Results from the whole-brain analysis for the contrast (tACS OFF > tACS 180°) for the 2-back task (Cluster corrected Z = 2.3, p < 0.05, n=21).

|  | | **Region** | **Cluster size** | **Z Peak** | **MNI (peak)** | | |
| --- | --- | --- | --- | --- | --- | --- | --- |
|  |  |  |  |  | **X** | **Y** | **Z** |
| **tACS 180° < tACS OFF** | | | | | | | |
|  | R | Precuneus cortex | 5664 | 4.60 | 4 | -56 | 14 |
|  | L | Precuneus cortex |  | 4.30 | -16 | -56 | 30 |
|  | L | Postcentral gyrus; Superior parietal lobule |  | 4.04 | -12 | -48 | 64 |
|  | L | Postcentral gyrus; Superior parietal lobule |  | 3.96 | -22 | -42 | 74 |
|  | R | Posterior cingulate cortex |  | 3.84 | 4 | -36 | 26 |
|  |  | Precuneus cortex |  | 3.82 | 0 | -58 | 18 |
|  | L | Frontal orbital cortex | 4856 | 4.85 | -28 | 34 | -10 |
|  | L | Anterior cingulate gyrus |  | 4.66 | -8 | 32 | -8 |
|  | L | Subcallosal cortex |  | 4.05 | -4 | 12 | -6 |
|  | L | Mammillary body |  | 3.95 | -2 | 0 | -14 |
|  | L | Frontal pole |  | 3.85 | -14 | 56 | 16 |
|  | L | Superior frontal gyrus |  | 3.78 | -22 | 38 | 52 |
|  | L | Inferior parietal lobule | 711 | 3.26 | -48 | -70 | 32 |
|  | L | Inferior parietal lobule |  | 3.20 | -44 | -70 | 32 |
|  | L | Inferior parietal lobule |  | 3.17 | -40 | -80 | 30 |
|  | L | Inferior parietal lobule |  | 2.99 | -44 | -66 | 40 |
|  | L | Inferior parietal lobule |  | 2.90 | -36 | -82 | 44 |
|  | L | Inferior parietal lobule |  | 2.87 | -46 | -78 | 36 |

For each cluster, the peak and 5 local maxima within the cluster are listed along with x-y-z locations in MNI space.

No clusters were observed for the contrast (tACS 180° > tACS OFF).

R = right hemisphere; L = left hemisphere

**Table supplement 3 (related to Figure 4B).** Results from the whole-brain analysis for the contrast (tACS 0° > tACS OFF) > (tACS 180° > tACS OFF) for the 2-back task (Cluster corrected Z = 2.3, p < 0.05, n=21).

|  | | **Region** | **Cluster size** | **Z Peak** | **MNI (peak)** | | |
| --- | --- | --- | --- | --- | --- | --- | --- |
|  |  |  |  |  | **X** | **Y** | **Z** |
|  | L | Superior frontal gyrus | 626 | 3.41 | -24 | 26 | 58 |
|  | L | Orbitofrontal cortex | 505 | 4.17 | -22 | 38 | -12 |
|  | L | Inferior parietal lobule | 399 | 3.28 | -44 | -66 | 38 |

No clusters were observed for the contrast (tACS 180° > tACS OFF) > (tACS 0° > tACS OFF)

R = right hemisphere; L = left hemisphere

**Table supplement 4 (related to Figure 4A).** Results from the whole-brain analysis for the contrast (tACS 0° > tACS OFF) for the CRT task (Cluster corrected Z = 2.3, p < 0.05, n=20).

|  | | **Region** | **Cluster size** | **Z Peak** | **MNI (peak)** | | |
| --- | --- | --- | --- | --- | --- | --- | --- |
|  |  |  |  |  | **X** | **Y** | **Z** |
| **tACS 0° > tACS OFF** | | | | | | | |
|  | R | Temporal occipital fusiform gyrus; Occipital fusiform gyrus | 1118 | 3.56 | 40 | -60 | -14 |
|  | R | Temporal occipital fusiform gyrus; Inferior temporal gyrus |  | 3.51 | 40 | -60 | -10 |
|  | R | Lateral occipital cortex (inferior division) |  | 3.43 | 36 | -82 | -6 |
|  | R | Occipital fusiform gyrus |  | 3.40 | 26 | -84 | -12 |
|  | R | Lateral occipital cortex |  | 3.34 | 42 | -74 | -2 |
|  | R | Lateral occipital cortex |  | 3.30 | 38 | -60 | -4 |
|  | L | Occipital fusiform gyrus; Occipital pole | 792 | 3.98 | -24 | -90 | -10 |
|  | L | Lateral occipital cortex (inferior division) |  | 3.73 | -30 | -90 | -10 |
|  | L | Temporal occipital fusiform gyrus; Occipital fusiform gyrus |  | 3.61 | -38 | -62 | -12 |
|  | L | Occipital fusiform gyrus |  | 3.57 | -36 | -66 | -10 |
|  | L | Occipital pole; Lateral occipital cortex |  | 3.33 | -32 | -90 | 8 |
|  | L | Temporal occipital fusiform gyrus; Occipital fusiform gyrus |  | 3.28 | -38 | -62 | -18 |
|  | R | Insular cortex | 572 | 3.56 | 44 | 10 | -6 |
|  | R | Precentral gyrus |  | 3.51 | 54 | 6 | 12 |
|  | R | Frontal orbital cortex; Insular cortex |  | 3.34 | 42 | 18 | -10 |
|  | R | Frontal operculum cortex |  | 3.01 | 46 | 14 | 4 |
|  | R | Inferior frontal gyrus, pars opercularis |  | 2.83 | 54 | 18 | 18 |
|  | R | Precentral gyrus |  | 2.74 | 68 | 8 | 8 |

For each cluster, the peak and 5 local maxima within the cluster are listed along with x-y-z locations in MNI space.

No clusters were observed for the contrast (tACS OFF > tACS 0°).

R = right hemisphere; L = left hemisphere

**Table supplement 5 (related to Figure 4A).** Results from the whole-brain analysis for the contrast (tACS 180° > tACS OFF) for the CRT task (Cluster corrected Z = 2.3, p < 0.05, n=20).

|  | | **Region** | **Cluster size** | **Z Peak** | **MNI (peak)** | | |
| --- | --- | --- | --- | --- | --- | --- | --- |
|  |  |  |  |  | **X** | **Y** | **Z** |
| **tACS 180° > tACS OFF** | | | | | | | |
|  | R | Cerebellum (vermis) | 656 | 3.69 | 4 | -68 | -20 |
|  | R | Lingual gyrus |  | 3.69 | 16 | -60 | -14 |
|  | L | Cerebellum (vermis) |  | 3.34 | -4 | -70 | -16 |
|  | R | Cerebellum |  | 3.30 | 20 | -58 | -18 |
|  | L | Lingual gyrus |  | 3.29 | -10 | -70 | -14 |
|  | R | Lingual gyrus |  | 3.21 | 12 | -70 | -14 |
|  | R | Occipital fusiform gyrus; Lateral occipital cortex | 632 | 3.68 | 30 | -88 | -14 |
|  | R | Occipital fusiform gyrus; Lateral occipital cortex |  | 3.66 | 32 | -82 | -10 |
|  | R | Lateral occipital cortex (inferior division) |  | 3.58 | 52 | -78 | 0 |
|  | R | Lateral occipital cortex (inferior division) |  | 3.56 | 28 | -82 | 2 |
|  | R | Lateral occipital cortex (inferior division) |  | 3.53 | 42 | -66 | 6 |
|  | R | Lateral occipital cortex (inferior division) |  | 3.31 | 36 | -76 | -2 |
|  | R | Supplementary motor cortex | 627 | 3.91 | 2 | -4 | 58 |
|  | L | Supplementary motor cortex |  | 3.72 | -6 | 6 | 48 |
|  | L | Superior frontal gyrus; Supplementary motor cortex |  | 3.09 | -10 | -6 | 66 |
|  | R | Paracingulate gyrus; Supplementary motor cortex |  | 2.86 | 10 | 8 | 48 |
|  | L | Paracingulate gyrus; Supplementary motor cortex |  | 2.76 | -14 | 8 | 40 |
|  | L | Lateral occipital cortex (inferior division) | 546 | 3.51 | -40 | -86 | -14 |
|  | L | Temporal occipital fusiform cortex |  | 3.47 | -26 | -62 | -18 |
|  | L | Occipital fusiform gyrus |  | 3.35 | -36 | -76 | -14 |
|  | L | Lateral occipital cortex (inferior division) |  | 3.02 | -48 | -82 | -12 |
|  | L | Temporal occipital fusiform cortex |  | 2.99 | -24 | -56 | -18 |
|  | L | Lateral occipital cortex (inferior division) |  | 2.98 | -44 | -76 | -2 |

For each cluster, the peak and 5 local maxima within the cluster are listed along with x-y-z locations in MNI space.

No clusters were observed for the contrast (tACS OFF > tACS 180°).

R = right hemisphere; L = left hemisphere

**Table supplement 6 (related to Figure 5).** Results from the PPI analysis of functional connectivity for synchronous and desynchronous tACS conditions for the 2-back task (Cluster corrected Z = 2.3, p < 0.05, n=21).

|  | | **Region** | **Cluster size** | **Z Peak** | **MNI (peak)** | | |
| --- | --- | --- | --- | --- | --- | --- | --- |
|  |  |  |  |  | **X** | **Y** | **Z** |
| **tACS 0° > tACS OFF (seed: IPL-electrode)** | | | | | | | |
|  | L | Dorsolateral prefrontal cortex;  Superior frontal gyrus | 458 | 3.35 | -30 | 48 | 28 |
| **tACS 0° > tACS OFF (seed: MFG-electrode)** | | | | | | | |
| No significant clusters | | | | | | | |
| **tACS 180° > tACS OFF (seed: IPL-electrode)** | | | | | | | |
|  | L | Lateral occipital cortex (superior division) | 561 | 3.24 | -36 | -90 | 14 |
|  | L | Inferior temporal gyrys; Middle temporal gyrus; Superior temporal gyrus | 468 | 3.68 | -42 | -60 | -6 |
|  | R | Lateral occipital cortex (superior division) | 525 | 3.76 | 36 | -84 | 22 |
|  | R | Superior temporal gyrus; Parietal Operculum | 476 | 3.26 | 60 | -38 | 20 |
| **tACS 180° > tACS OFF (seed: MFG-electrode)** | | | | | | | |
|  | L | Lateral occipital cortex (superior division); Middle temporal gyrus; Superior temporal gyrus | 1767 | 3.81 | -42 | -60 | -4 |
|  | L | Lateral occipital cortex (inferior division) | 55 | 3.16 | -54 | -72 | -18 |
|  | R | Lateral occipital cortex (superior division) | 671 | 3.55 | 30 | -72 | 18 |

No clusters were observed for the contrasts tACS OFF > tACS 0°/180°

R = right hemisphere; L = left hemisphere
